# Supplementary material for: Maize brace roots provide stalk anchorage
Source: Plant Direct. 2020 Nov 8;4(11):e00284. doi: 10.1002/pld3.284 (PMC7649601; doi:10.1002/pld3.284)
Supplement: Supplementary file 1 — Fig S1‐S5 [file PLD3-4-e00284-s001.pdf]

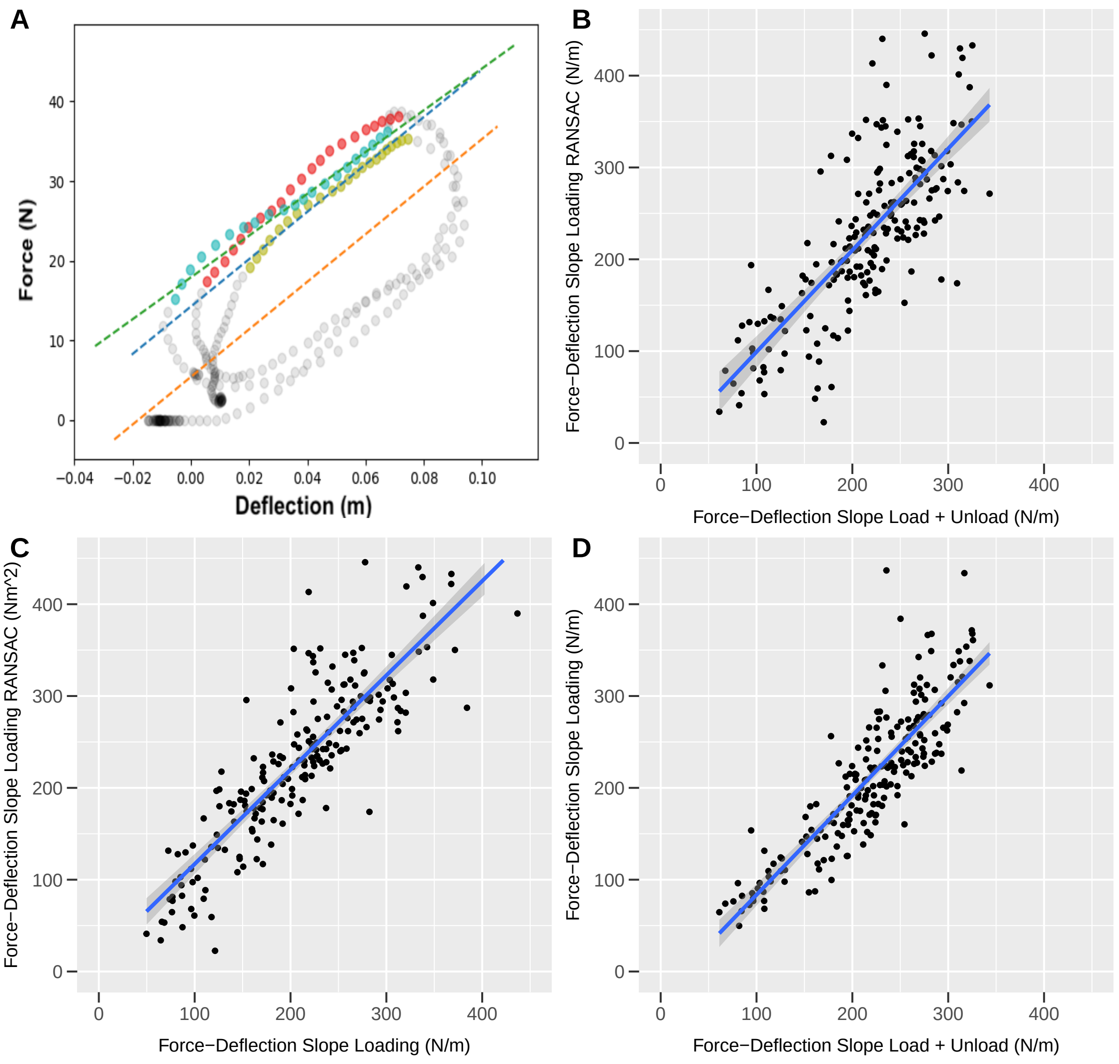

Figure S1: Comparison of Slope Extraction Methods. (A) Force-Deflection data was plotted for each test. In this representative image, the three cycles (different colors) that have been identified as loading due to the monotonically increasing deflections are shown by solid dots and grey dots represent unloading data. Dashed lines represent the three different slope fitting approaches: blue: RANSAC to the longest consecutive loading curve; green: line fit to all three loading curves; orange: line fit to loading and unloading data. (B) Correlation between the Force-Deflection slopes extracted by RANSAC fit to the loading data only and a line fit to loading and unloading data ($r = 0.86$, $p < 2.2E^{-16}$). (C) Correlation between the Force-Deflection slopes extracted with the two loading methods ($r = 0.87$, $p < 2.2E^{16}$). (D) Correlation between the Force-Deflection slopes extracted by a line fit to the loading data only and a line fit to loading and unloading data ($r = 0.76$, $p < 2.2E^{-16}$). Lines in B, C and D represent generalized linear model (glm) fit and shading indicates a 95% confidence interval.

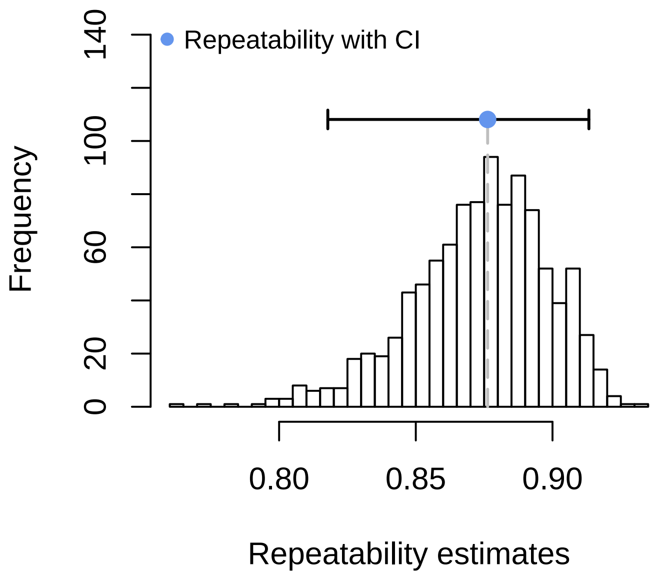

Figure S2: Measurements of the Force-Deflection Slope have High Repeatability. Repeatability analysis for plants in Figure 1 were calculated using the rptR package in R and confidence intervals determined by bootstrapping (n = 1000). The mean repeatability (R) was 0.876 with a 0.025 SE and $p = 2.31E^{-49}$.

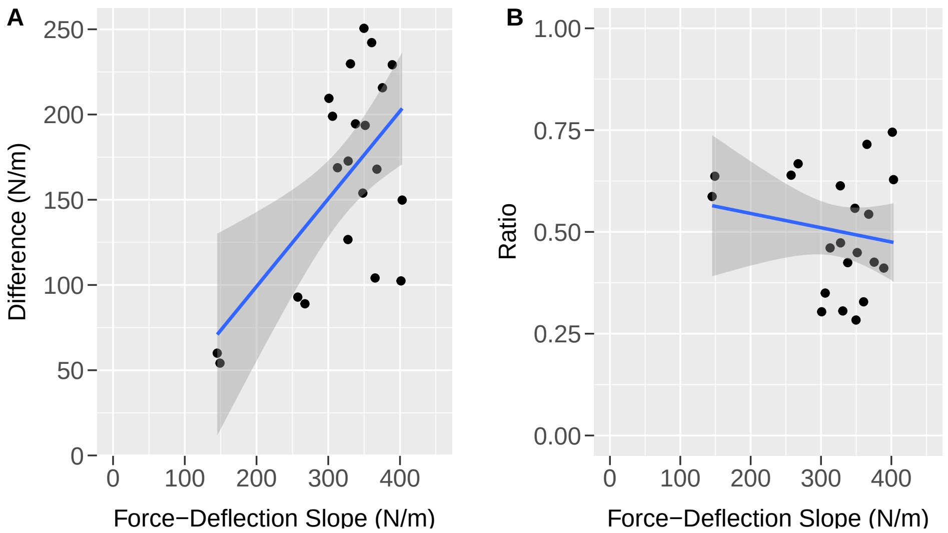

Figure S3: The Initial Force-Deflection Slope is Correlated with the Absolute, but Not the Relative Contribution of Brace Roots. (A) Comparison of the Force-Deflection slope with brace roots intact and the difference in Force-Deflection slope after all brace roots have been removed. There is a positive correlation (r = 0.59, p = 0.004) between these measurements. As the difference relies on the initial Force-Deflection slope, this correlation is to be expected. (B) Comparison of the Force-Deflection slope with brace roots intact and the ratio of Force-Deflection slope after all brace roots have been removed. There is no correlation (r = -0.17, p = 0.461) between these measurements. Lines represent generalized linear model (glm) fit and shading indicates a 95% confidence interval.

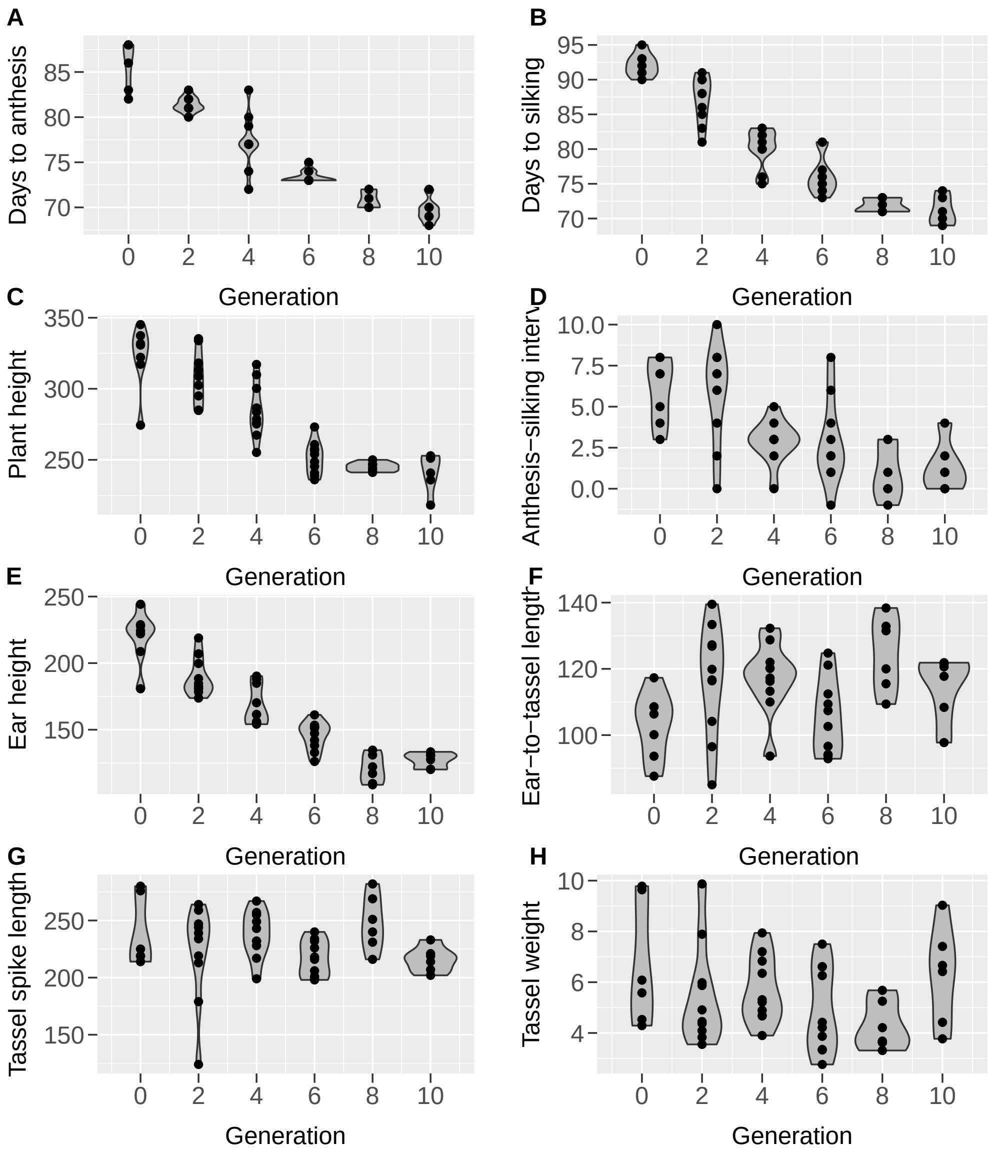

Figure S4: The subset of Tusón lines selected for this study show a reduction (A) days to anthesis, (B) days to silking, (C) plant height, (D) anthesis-silking interval, and (E) ear height. There was no change in (F) ear-to-tassel length, (G) tassel spike length, or (H) tassel dry weight in this subset of lines. Data from the Delaware grow out of these lines as published in Teixeira et al. [2015].

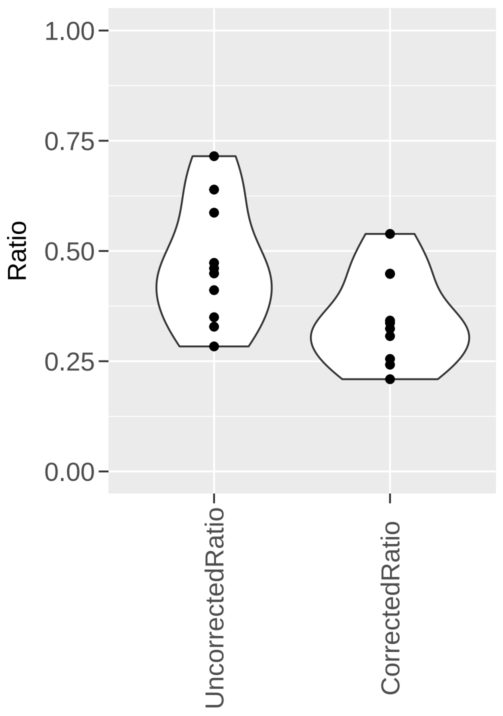

Figure S5: Correcting for the change in effective beam length increases the relative contribution of brace roots. We manually measured the attachment height of brace roots for the CML258 plants in Plot A, which ranges between 0.015m to 0.032m. When accounting for this change in length, and calculating the ratio contribution of brace roots, the effect of brace roots increases rather than decreases.
